# Hypothetical model of perceived adherence to treatment among patients with coronary heart disease after a percutaneous coronary intervention

**DOI:** 10.1002/nop2.381

**Published:** 2019-09-27

**Authors:** Outi Kähkönen, Päivi Kankkunen, Terhi Saaranen, Heikki Miettinen, Helvi Kyngäs

**Affiliations:** ^1^ Research Unit of Nursing Science and Health Management University of Oulu Oulu Finland; ^2^ Department of Nursing Science University of Eastern Finland Kuopio Finland; ^3^ Kuopio University Hospital Kuopio Finland

**Keywords:** adherence to treatment, coronary heart disease, nursing theory, perceived health, percutaneous coronary intervention, social support

## Abstract

**Aim:**

To test the hypothetical model of adherence to treatment among patients with coronary disease after percutaneous coronary intervention.

**Design:**

A descriptive, explanatory, cross‐sectional survey.

**Methods:**

The study was conducted in 2013 with 416 patients in five hospitals in Finland. The adherence of patients with chronic disease instrument, the adherence visual analogue scale, the social support for people with coronary heart disease instrument, the EuroQoL five‐dimensional scale and EuroQoL visual analogue scale were used. The data were analysed using descriptive statistic. The hypothetical model was tested using structural equation modelling.

**Results:**

The hypothetical model explained 30% of perceived adherence to treatment. Structural equation modelling confirmed that motivation, support from physicians and next of kin had direct associations with adherence. Indirectly, informational support, results of care, perceived health, anxiety and depression were associated with adherence. The background variables associated with adherence were gender, relationship, physical activity, consumption of vegetables and consumption of alcohol.

## 1 INTRODUCTION

Coronary heart disease (CHD) is responsible for the largest proportion of premature mortality worldwide (World Health Organization, 2018) although advanced treatment therapies have significantly enhanced cardiac patients’ prognoses (Neumann et al., 2018). According to the WHO (2018), about 75% of mortality would be avoided with better adherence to treatment, which is a major element in improving patients’ prognosis and decreasing the progression of CHD after a percutaneous coronary intervention (PCI) (Booth et al., 2014; Neumann et al., 2018). However, the failure to make lifestyle changes and misunderstandings regarding CHD and a cardioprotective lifestyle are common (Perk et al., 2015; Wiecek et al., 2019). The predominance of non‐adherence regarding medication (Ibanez et al., 2017; Pettersen et al., 2018) and a healthy lifestyle (Booth et al., 2014; Ibanez et al., 2017) is estimated to range from 30% to 60% among patients with CHD after PCI (Perk et al., 2015).

The use of PCI has increased steadily over the past decade. When the treatment is successful, a patient may even be discharged on the same day as the procedure. Shorter hospitalizations are clearly cost‐effective (Rubimbura et al., 2019; Shroff et al., 2016), but the responsibility for care transfers quickly to patients, which may lead to a diminished understanding of the seriousness of CHD and the lifelong chronic nature of the disease (Ibanez et al., 2017).

### 1.1 Background

The Theory of Adherence among People with Chronic Disease (1999) is originally developed by Kyngäs (1999) and developed further among different patient groups (Kähkönen et al., 2015; Oikarinen, Engblom, Kääriäinen, & Kyngäs, 2017). According to this theory, adherence to treatment is defined as an active, intentional and responsible process of care where patients with chronic conditions work to maintain their health in collaboration with healthcare professionals. Adherence to treatment consists of adherence to medication and a healthy lifestyle, including a healthy diet, physical activity, non‐smoking and moderate alcohol consumption. Explanatory factors associated with adherence to treatment according to Kyngäs's original Theory of Adherence among People with Chronic Disease are responsibility, cooperation, a sense of normality, motivation, the results of care, support from next of kin, support from nurses, support from physicians and a fear of complications (Kähkönen et al., 2018; Kyngäs, 1999; Oikarinen et al., 2017). The Theory of Adherence among People with Chronic Disease as a theoretical framework to evaluate post‐PCI patients’ adherence has been tested. Cooperation, a sense of normality, motivation, the results of care, support from next of kin, support from physicians and a fear of complications have been shown to explain adherence to treatment among patients with CHD after PCI (Kähkönen et al., 2018). Post‐PCI patients’ non‐adherence to treatment may be intentional or unintentional. Intentional non‐adherence is associated with the patient's conscious decision to stop taking the prescribed medicines, reduce the dosage of one's medication or neglect treatment recommendations. The reasons for intentional non‐adherence can be numerous such as financial obstacles or medication side effects. In contrast, unintentional non‐adherence refers to a patient's lack of capacity or cognitive resources, which can lead to non‐adherence. (Molloy et al., 2014; Pettersen et al., 2018).

Certain psychological factors, such as poor perceived health (De Smedt et al., 2016; Sajobi et al., 2018) and a lack of social support (Valtorta, Kanaan, Gilbody, Ronzi, & Hanratty, 2016; Xia & Li, 2018), are evidently associated with the prognosis of CHD (De Smedt et al., 2016; Sajobi et al., 2018; Valtorta et al., 2016). Perceived health reflects an individual's overall and subjective perceptions of his or her own health status, which include physical and psychological aspects (De Smedt et al., 2014). Perceived health predicts mortality and morbidity (Sajobi et al., 2018) despite the manifestation of other co‐morbidities and risk factors among patients with CHD. Hence, perceived health is an important outcome measure among patients with CHD. The significance of patients’ subjective experience of their own health status in clinical practice in undeniable (De Smedt et al., 2016; Sajobi et al., 2018).

Furthermore, a low level of social support is considered a risk factor for CHD in healthy people, as well as a risk factor for worse prognoses and higher mortality among patients diagnosed with CHD (Valtorta et al., 2016; Xia & Li, 2018). The dimensions of social support, such as support from next of kin, physicians and nurses, have been proven to be significant factor associated with adherence among chronically ill patients (Kähkönen et al., 2018; Kyngäs, 1999). Social support has been defined as a dynamic interpersonal process centred on the reciprocal exchange of information. Social support changes across contexts, and it is manifested between providers and recipients. Social support may appear multifaceted depending on its context. (Brooke & Collins, 2015). In particular, the importance of social support is clear during a stressful life event, such as an acute cardiac event. Social support provides security, a feeling of love and a community spirit (Aazami, Jaafarpour, & Mozafari, 2016).

Adherence to treatment (Booth et al., 2014; Perk et al., 2015), especially adherence to medication (Booth et al., 2014; Brown et al., 2014; Pettersen et al., 2018; Reuter et al., 2015), has been of interest in many studies. Furthermore, perceived health (De Smedt et al., 2016) and social support (Valtorta et al., 2016) are widely studied. These concepts are evidently associated the prognoses of patients with CHD (De Smedt et al., 2016; Valtorta et al., 2016). Although adherence to treatment, perceived health and social support have been widely studied, an absence of previous research on the relationships between these theoretical concepts is undeniable. In fact, CHD is a major public health problem worldwide, causing a significant financial and human burden. Therefore, improved knowledge is needed to increase our theoretical understanding of adherence to treatment and associated factors to develop effective nursing interventions that meet current treatment needs.

## 2 THE STUDY

### 2.1 Aim

**Figure 1:**
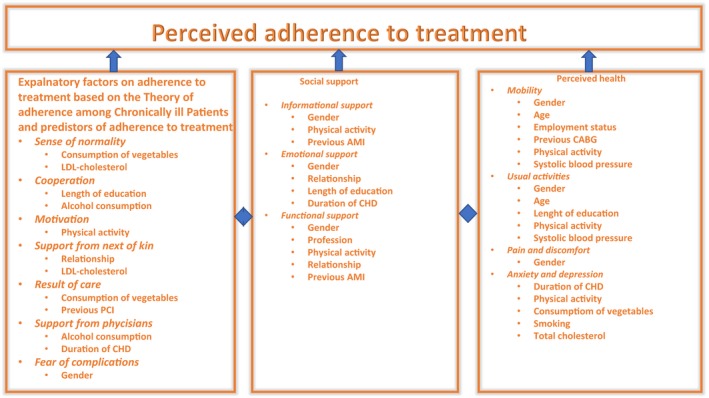
Hypothetical model of perceived adherence to treatment and related factors. *Abbreviations*: AMI, acute myocardial infarction; CABG, coronary artery bypass crafting; CHD, coronary heart disease; LDL, low‐density lipoprotein; PCI, percutaneous coronary intervention

The aim of the present study was to test whether empirical data would fit the proposed hypothetical model of perceived adherence to treatment (Figure 1) based on four sub‐studies: testing the theory of adherence to treatment of chronically ill patient among patients (Kähkönen et al., 2015), description and exploration of the predictors of adherence to treatment (Kähkönen et al., 2018), perceived health (Kähkönen, Saaranen, Lamidi, Miettinen, & Kankkunen, 2017), received social support (Kähkönen, Kankkunen, Miettinen, Lamidi, & Saaranen, 2016) and associated factors among patients with CHD after PCI. The hypothetical model of perceived adherence to treatment was created based on the statistically significant findings in these sub‐studies. The major hypothesis of this study was that the hypothetical model of perceived adherence to treatment is suitable for explaining adherence to treatment and associated factors among patients with CHD after PCI. To test the hypothetical model of perceived adherence to treatment, the following specific hypotheses were set:

1. Based on the tested Theory of Adherence of Patients with Chronic Disease among post‐PCI patients: sense of normality, cooperation, motivation, support from next of kin, results of care, support from physicians and fear of complications predict perceived adherence to treatment.
2. Dimensions of social support: informational, support and functional support predict perceived adherence to treatment.
3. Perceived health and its dimension: problems in mobility, usual activities, pain, discomfort, anxiety and depression predict perceived adherence to treatment.
4. Sociodemographic (gender, age, relationship, length of education, employment status, profession), health behavioural (consumption of vegetables, alcohol consumption, physical activity, smoking) and disease‐specific factors (LDL cholesterol, total cholesterol, systolic blood pressure, duration of CHD, pervious PCI, AMI or CABG) predict with perceived adherence to treatment.

### 2.2 Design

This is the combination study which tests the hypothetical model based on the results of descriptive, explanatory, cross‐sectional surveys regarding the level of adherence to treatment, received social support and perceived health of patients with CHD after PCI.

### 2.3 Participants

A convenience sample of patients with CHD was recruited into the study 4 months after PCI, when the patients had recovered from treatment and adapted to everyday life. Patients were drawn from a multicentre arrangement including two university hospitals and three central hospitals in Finland. The inclusion criteria were an age of 18–75 years, PCI having been conducted in under elective or acute circumstances and fluency in the Finnish language. Patients diagnosed with a memory disorder were excluded from the study. The potential participants’ suitability for participation was evaluated, and 572 patients met the inclusion criteria and received verbal and written information about the study from the registered nurse of the medical ward during the hospitalization. After that 91% (520) agreed to participate and provided informed consent. According to power analyses, this sample size was large enough to detect statistical significance with a power of 80% and a significance level of 0.05 given relatively small correlations (0.14). This number of observations and incidence rate can detect about 7%–13% of the difference between the groups.

### 2.4 Data collection

The data were collected using a postal questionnaire 4 months after PCI between January and December 2013 via the following four instruments:

1. The adherence of patient with chronic disease (ACDI) instrument, which is based on a theoretical model of chronically ill patients developed and tested originally by Kyngäs (1999). The ACDI contained 37 items of adherence to treatment, which were rated on a 5‐point Likert scale (‘definitely disagree’ to ‘definitely agree’).
2. The adherence visual analogue scale (A‐VAS) instrument, which records the respondents’ self‐rated adherence to treatment from the best imaginable adherence to treatment (100) to the worst imaginable adherence to treatment (0) developed by Kähkönen et al. (2018).
3. The social support for people with coronary heart disease (SSCHD) instrument, developed by Kähkönen et al. (2016) for the present study and based on the Cohen and Wills's (1985) theory of social support. The SSCHD included 14 items related to receiving social support: informational, emotional and functional support, which were rated on a 5‐point Likert scale (‘definitely disagree’ to ‘definitely agree’).
4. The EuroQoL five‐dimensional scale (EQ‐5D‐5L) regarding the severity of problems on the perceived health dimensions were rated on a 5‐point Likert scale (1 = no problems, 2 = mild problems, 3 = some problems, 4 = moderate problems, 5 = extreme problems).
5. The EuroQoL visual analogue scale (EQ‐VAS) ranking respondents’ perceived health with endpoints labelled the best imaginable health (100) and the worst imaginable health state (0).

Additionally, the questionnaire included 18 self‐reported background questions related to demographic details (age, gender, relationship, profession, employment status and length of education), disease‐specific information (duration of CHD, previous AMI, previous PCI, previous CABG, systolic and diastolic blood pressure, total cholesterol and LDL cholesterol) and health behaviour (physical activity, consumption of vegetables, smoking habits and alcohol consumption). In summary, the questionnaire consisted of 56 items, which were rated on a 5‐point Likert scale, two visual analogue scales (VAS) ranking 0–100 and 18 background questions.

Of the recruited patients with CHD after a PCI, 80% (*n* = 418) completed the study. Two questionnaires were rejected because they were inadequately completed and had missing values.

### 2.5 Ethical considerations

The statement of the Ethical Review Board of the university hospital (ref. 74//2012) and the approval for the study were obtained from each research centre separately. The research was conducted following the ethical principles of the Finnish Advisory Board on Research Integrity (2018) and the World Medical Association (2013). Patients provided their informed consent prior to being discharged. Participants signed informed consent forms after receiving verbal and written information about the study from a registered nurse in accordance with the Declaration of Helsinki. The participants were told about the voluntary nature of the participation and their option to withdraw at any point. Furthermore, the contact details of the researchers were given to the participants in case they had any additional questions. Additionally, participants were informed about the confidential nature of the research. The researcher collected the data via the postal questionnaire 4 months after PCI and analysed the data confidentially, using codes instead of respondent names (Finnish Advisory Board on Research Integrity, 2018; World Medical Association, 2013).

### 2.6 Data analysis

**Figure 2:**
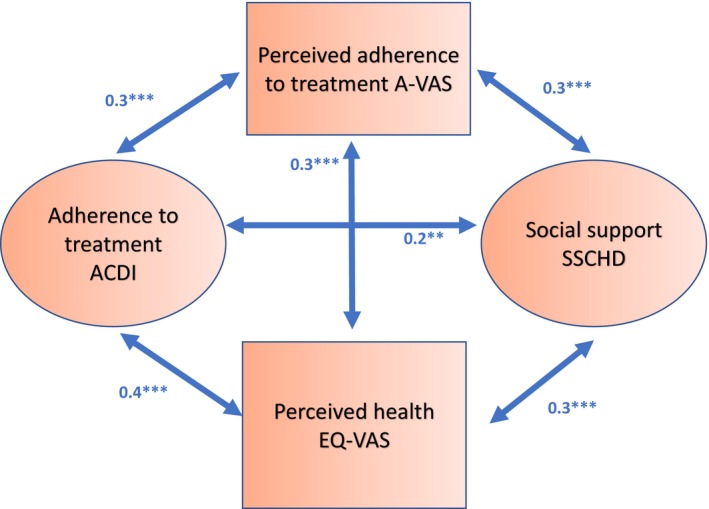
Testing covariances and correlations between the instruments used in the study. *Note*: *p < .05, **p < .01, ***p < .001. *Abbreviations*: ACDI, adherence of people with chronic disease instrument; A‐VAS, adherence visual analogue scale; EQ‐VAS, EuroQoL visual analogue scale; SSCHDI, social support for people with coronary heart disease instrument

The Statistical Package for Social Sciences (SPSS 21) software for Windows and Analysis of Moment Structures (AMOS) Version 21 were used to analyse the data. The phenomena that had a positive effect on patients’ prognoses were of the interest in this study. At the beginning of the analysis, the correlations between the instruments used in sub‐studies I‐IV were examined using structural equation modelling (*SEM*) because these specific phenomena and the relationships between the involved concepts have not previously been studied together. (Kellar & Kelvin, 2013). Statistically significant associations between the ACDI, A‐VAS, EQ‐VAS and SSCHDI instruments were found and these are presented in Figure 2. Therefore, it can be assumed that these instruments can be used to evaluate perceived adherence to treatment among post‐PCI patients. Abbreviations: ACDI = adherence of people with chronic disease instrument; EQ‐VAS = EuroQoL visual analogue scale; SSCHDI = social support for people with coronary heart disease instrument; A‐VAS = adherence visual analogue scale.

The premise of the hypothetical model based on the results in the sub‐studies I‐IV. These sub‐studies confirmed that the Theory of Adherence of People with Chronic Disease is a suitable theoretical framework of the research for patients with CHD after PCI (sub‐study I Kähkönen et al., 2015). According to the theory, cooperation, a sense of normality, motivation, the results of care, support from next of kin, support from physicians and a fear of complications are associated with adherence to treatment among patients with CHD after PCI (Kähkönen et al., 2018). Based on the sub‐study III, perceived health was decreased among patients with CHD after PCI compared with Finnish population norms. Most commonly respondents reported pain and discomfort, problems in mobility, or anxiety and depression. (Kähkönen et al., 2017). Sub‐study IV indicated that informational support was the strongest form of social support and functional support was the weakest. Although respondents received high information regarding CHD and self‐care, there was discrepancy regarding respondents’ health behaviour and secondary prevention target values. The information regarding physical exercise and rehabilitation were the weakest forms of informational support. Peer support was the lowest form of emotional support. Regarding functional support, the weakest form was respondents’ perceived care about their coping by healthcare professionals. (Kähkönen et al., 2016).

In the next step, the hypothetical model of perceived adherence to treatment was constructed based on the follows statistically significant factors in the sub‐studies I‐IV: sense of normality, cooperation, motivation, support from next of kin, results of care, support from physicians and fear of complications (sub‐study I‐II; Kähkönen et al., 2015, 2018), dimension of perceived health: mobility, usual activities, pain and discomfort, anxiety and depression (sub‐study III; Kähkönen et al., 2017) and the dimension of social support: informational, emotional and functional support (sub‐study IV; Kähkönen et al., 2016), (Figure 1). Additionally, the following background factors were hypothesized to be the predictors of perceived adherence to treatment based on the sub‐studies I‐IV: sociodemographic factors (age, gender, relationship, length of education, profession, employment status); health behavioural factors (smoking, physical activity, vegetable consumption, alcohol consumption); and disease‐specific factors (systolic blood pressure, diastolic blood pressure, LDL cholesterol, previous CABG, previous PCI, previous AMI and duration of CHD: Kähkönen et al., 2016; Kähkönen et al., 2015; Kähkönen et al., 2017).

The correlation between the hypothesized model of perceived adherence to treatment and the observed correlation matrix was examined using *SEM*. The chi‐square test and its derivatives, CFI and RMSEA were used to estimate the adequacy of the model. A weak effect was indicated by values <0.10, a medium effect was indicated by values ~0.30, and a major effect was indicated by values >0.5. (Shreiber, Amaury, Stage, Barlow, & King, 2006; Bollen, Harden, Ray, & Zavisca, 2014; Zhang, 2017).

### 2.7 Validity, reliability and rigour of instruments

The construct validity of ACDI and SSCHD was verified with an exploratory factor analysis (EFA). In the ACDI, the alpha coefficients ranged from 0.40 to 0.88 and the alpha coefficient of the entire scale was 0.80, indicating sufficient‐to‐high internal consistency (Polit & Beck, 2016) as seen also in the earlier studies (Oikarinen et al., 2017; Ylimäki, Kanste, Heikkinen, Bloigu, & Kyngäs, 2015). Regarding ACDI, eleven factors explained 65% of the total variance, communalities varied between 0.20 and 0.80 and the factor loadings were between 0.30 and 0.90 (Polit & Beck, 2016).

A‐VAS was developed for the present study. Therefore, the data of validity and reliability were not available. *SEM* is valuable data analysis tool in testing theories and in evaluating construct validity of the instruments (Kellar & Kelvin, 2013). Therefore, the validity was tested between the A‐VAS and the adherence items in the ACDI (adherence to medication and adherence to a healthy lifestyle), which has been used widely measuring adherence among different patient groups. ACDI is based on the theory of adherence to treatment among chronically ill patients (Kyngäs, 1999). A statistically significant correlation using *SEM* was found between A‐VAS and adherence items in the ACDI (0.50).

Additionally, EFA was conducted to verify the construct validity of SSCHD instrument and it produced a factor solution where three factors explained 59.3% of the total variance and communalities in all items were good (>0.30) and the factor loading of all variables was 0.30–0.87. Cronbach's alpha values varied acceptability levels (0.60–0.90), indicating sufficient‐to‐ high internal consistency. The alpha coefficient of the SSCHD instrument was 0.78 indicating satisfactory internal consistency. (Polit & Beck, 2016).

The EQ‐5D instrument is a widely used, standardized and validated generic instrument for measuring perceived health. In the earlier studies, the EQ‐5D appears to have good convergent and known‐groups validity (Yfantopoulos & Chantzaras, 2017).

Furthermore, the questionnaire's face validity was evaluated by three nurses who had extensive experience nursing cardiac patients and 15 patients with CHD after PCI. Based on their feedback, changes were made to increase the questionnaire's intelligibility and usability.

## 3 RESULTS

The study participants were mainly male CHD patients (75.5%, *N* = 314) aged from 18 to 75, with a mean age of 63.2 years (range 38–75, *SD* 8). Most patients were married or in a close relationship. Respondents reported high levels of adherence to treatment (mean = 89.9, median = 90.0, range 30.0–100.0, *SD* 11.2). The characteristics of the participants have been presented in detail previously by Kähkönen et al. (2018).

In the first phase, the direct positive association between adherence to treatment and the hypothetical model of perceived adherence to treatment was tested (Figure 1). The model included the predictors that were found statistically significant in the previous studies (Kähkönen et al., 2018), such as sense of normality, cooperation, motivation, support from next of kin, results of care, support from physicians and fear of complications (Kähkönen et al., 2018). Additionally, the model included the dimensions of social support (informational, emotional and functional support) (Kähkönen et al., 2016) and the dimensions of perceived health (mobility, usual activities, pain and discomfort, anxiety and depression) (Kähkönen et al., 2017). In addition, the model contained statistically significant background variables associated with adherence to treatment, social support and perceived health (age, gender, relationship, length of education, profession, employment status, smoking, physical activity, vegetable consumption, alcohol consumption, systolic blood pressure, diastolic blood pressure, LDL cholesterol, previous CABG, previous PCI, previous AMI and duration of CHD) (Kähkönen et al., 2016, 2017). These factors were hypothesized to be predictors of adherence to treatment.

Testing the hypothetical model of perceived adherence to treatment in the first phase indicated direct positive relationships between adherence to treatment and motivation, support from physicians, support from next of kin, perceived health and pain/discomfort. The model was rejected because the regression weights and the model fit were insignificant regarding the relationships between sense of normality, cooperation, results of care, fear of complications, mobility, self‐care, usual activities, anxiety/depression, emotional support and functional support. The estimates were as follows: *χ* ^2^ = 591.9, *df* = 127, *p* < .001, *χ* ^2^/*df* = 4.7, CFI = 0.8 and RMSEA = 0.9, indicating that the hypothesized model did not fit the empirical data.

**Figure 3:**
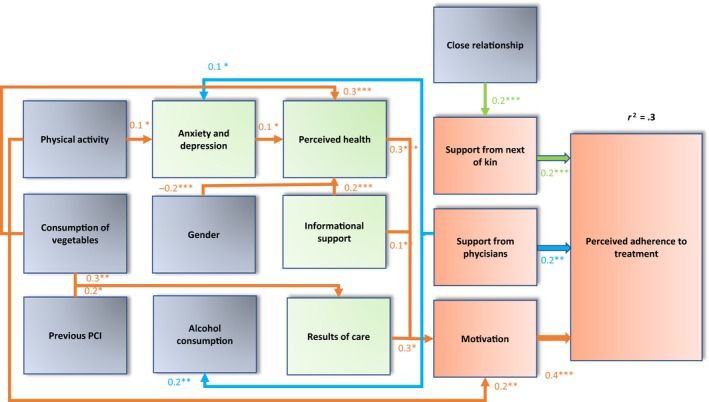
Testing the hypothetical model including the factors directly and indirectly associated with adherence to treatment among patients with coronary heart disease after percutaneous coronary intervention. The effects of the standardized estimates were evaluated as follows: the estimates had a weak effect if the values were <0.10, medium effect if the values were ~0.30 and they had a major effect if the values were >0.5. Covariances: informational support–support from physicians***, physical activity—informational support*, consumption of vegetables—physical activity***, consumption of vegetables—gender*** (Note: *p < .05, **p ≤ .01, ***p ≤ .001). (Shreiber et al., 2006)

In the eventual model (Figure 3), the strongest association was found between perceived adherence to treatment‐associated and motivation. Additionally, perceived adherence to treatment was directly associated with support from physicians and next of kin. An indirect but statistically significant association was found between perceived adherence to treatment and informational support, the results of care and perceived health, as measured via motivation. Furthermore, anxiety and depression and perceived health had a statistically significant association. Regarding background variables, indirect associations were found as follows: the close personal relationship was associated with support from next of kin, support from physicians was associated with alcohol consumption and anxiety and depression, previous PCI and consumption of vegetables were associated with the perceived results of care, male gender was associated with better perceived health and physical activity was associated with motivation and anxiety and depression. Nevertheless, the outlined structural equation model with standardized estimates was statistically significant, with the following estimates: *χ* ^2^ = 908.1, *df* = 365, *p* < .001, *χ* ^2^/*df* = 2.5, CFI = 0.8 and RMSEA = 0.6. Therefore, variables with ≥4 items were analysed using a unidimensional test. Considering the results, one item (16) was removed from the mean sum variable support from next of kin. In consequence, the outlined structural equation model indicated an acceptable model fit, with the following standardized estimates: CFI = 0.9 and RMSEA = 0.6. The chi‐square test results were as follows: *χ* ^2^ = 786.7, *df* = 338, *p* < .001 and *χ* ^2^/*df* = 2.4. The standardized path coefficients indicated a medium effect (_~_ 0.2–0.4) regarding the factors directly affecting adherence to treatment. (Shreiber et al., 2006; Bollen et al., 2014). The model explained 30% of the variance in the factors associated with adherence to treatment.

## 4 DISCUSSION

According to our knowledge, the theoretical model of perceived adherence has not been tested previously among post‐PCI patients. An absence of previous research on the relationships between the theoretical basis of perceived adherence to treatment is undeniable, although perceived health, social support and adherence to treatment have been widely studied. The present study indicated direct associations with perceived adherence to treatment and motivation, support from physicians and next of kin.

The strongest predictor to perceived adherence was motivation, whereupon it is essential to examine the factors underlying motivation to develop the nursing interventions needed to support patients’ adherence to proper medical treatment and a healthy lifestyle, which are the cornerstones of a good prognosis of CHD after PCI (Ghisi 2015; Ibanez et al., 2017; Shroff et al., 2016). The results of the present study confirmed that perceived health and results of care predicted motivation. Therefore, patients should know and understand the special characteristic of CHD and the chronic long‐term nature of CHD after PCI, which can help him or her recognize the importance of the results of care and perceived health. This requires informational support, which was also associated with motivation. These findings emphasize the importance of person‐centred care, which necessitates collaboration between practitioners and patients, as well as the patients’ expert participation in their own care. Additionally, physical activity was a predictor to motivation, as were anxiety and depression negatively. The benefits of physical activity are strongly documented in that it reduces modifiable CHD risk factors, such as hypercholesterolaemia, hypertension, overweight and stress, thus improving patients’ prognoses (Perk et al., 2015). Therefore, it is reasonable to emphasize the importance of patient education regarding physical activity so that patients are informed about its value (Edward et al., 2016; Ghisi et al., 2015; Perk et al., 2015).

Support from physicians was also directly associated with perceived adherence to treatment. At present, health care in Finland is undergoing a significant structural change, which is strongly associated with a reassessment of duties, including the transfer responsibility from physicians to nurses concerning chronically ill patients’ care paths (Health & social services reform, Ministry of Social Affairs & Health: The Government's key projects, 2017). However, based on the results of this study, during the acute phase after PCI, physician support is predictor of adherence to treatment. The therapeutic relationship between patients and physicians is important and the resources needed for this relationship should be guaranteed, as Du et al. (2016) have confirmed: cardiologist‐coordinated intensive follow‐up programmes have decreased cardiovascular risk factors, reduced medical costs, promoted adherence to medication and improved the long‐term prognoses of patients after PCI.

Furthermore, one important finding was that support from the next of kin was associated with perceived adherence to treatment. This result emphasizes the importance of the next of kin's participation in a patient's care and education. The goal of the next of kin should be to support and encouragement that is in accordance with the patient's wishes. Risk factors for CHD are generally associated with health behaviours, such as diet and physical activity. Adherence to a healthy lifestyle requires changes in health behaviours after PCI, which will be easier to make if the next kin understands the importance of adherence to a healthy lifestyle and supports the patient. Being married or in a close relationship was associated with receiving support from the next of kin. (Lammintausta et al., 2014). This is noteworthy because marriage or being a close relationship, which enables patients’ feelings of love, belonging to a community and security in life, has a protective effect on CHD patients’ prognoses according to prior evidence (Xia & Li, 2018). Hence, single patients are important to take into consideration and it is important to provide other forms of support, such as peer support, if social networks are limited (Guo & Harris, 2016; Xia & Li, 2018).

### 4.1 Limitations

This study includes certain theoretical and methodological limitations. Although perceived health, social support and adherence to treatment have been widely studied, an absence of previous research on the relationships between these main concepts complicated the theoretical basis for this study. Additionally, the fragmentation of these concepts was challenging in terms of comparing various studies. This fragmentation may also cause some significant aspects of the phenomena to go unidentified.

In terms of methodological limitations, the hypothetical model which was tested in this study may have some limitations regarding causality and generalizability. However, it is not the main concern in the present study, because the hypothetical model based on the three sub‐studies, which were verified previously (Kähkönen et al., 2016, 2015, 2018, 2017). However, A‐VAS was developed for this study and the reliability and validity of this instrument have not been tested previously, which could be a limitation in this study.

Regarding the questionnaire, the number of the total question was quite high, which could have been a burden to some patients and influencing the accuracy of the answers provided by the patients. Although the response rate was good (80%), this may be a limitation.

Finally, in self‐reported data collection methods, there is always a risk of the social desirability effect, where patients provide answers, they think are favourable instead of saying what they do or think (Althubaiti, 2016).

## 5 CONCLUSION

The tested hypothetical model was confirmed in part in this study. First, the Theory of Adherence among People with Chronic Disease is an applied suitable theoretical framework for evaluating adherence to treatment among patients with CHD after PCI: motivation, support from physicians and support from next of kin were the strongest predictors of perceived adherence to treatment and results of care predicted adherence indirectly. Instead, sense of normality, cooperation and fear of complication were not associated with perceived adherence to treatment. Second, perceived health and its dimension anxiety/depression were indirect predictors to perceived adherence to treatment. Other dimensions of perceived health: mobility, usual activities and pain/discomfort were not associated with perceived adherence to treatment. Third, informational support as a dimension of social support predicted perceived adherence to treatment via motivation, but emotional support and functional support were not associated with perceived adherence to treatment. Regarding background factors, physical exercise was predicted motivation and was associated with lower anxiety/depression. The close relationship was associated with support from next of kin. Instead, gender and consumption of vegetables via perceived health, alcohol consumption via support from physicians, prior PCI and consumption of vegetables via results of care predicted perceived adherence to treatment indirectly. Healthcare professionals were an important factor in adherence to treatment, especially among women, those who were physically inactive, those with low vegetable consumption and those without relationships. In the future, multidisciplinary studies are needed to develop evidence‐based interventions to support patient adherence to treatment.

## CONFLICT OF INTEREST

No conflict of interest has been declared by the authors.

## AUTHOR CONTRIBUTIONS

OK made substantial contributions to conception and design, or acquisition of data, or analysis and interpretation of data; OK, PK, TS, HM, PT, HK involved in drafting the manuscript or revising it critically for important intellectual content; Given final approval of the version to be published. Each author should have participated sufficiently in the work to take public responsibility for appropriate portions of the content; Agreed to be accountable for all aspects of the work in ensuring that questions related to the accuracy or integrity of any part of the work are appropriately investigated and resolved.

## FUNDING INFORMATION

The present study was supported by an educational grant from the Finnish Foundation of Cardiovascular Disease (16.4.2012) and Finnish Nursing Associations (6.6.2014).
